# Semi-automatic algorithm for construction of the left ventricular area variation curve over a complete cardiac cycle

**DOI:** 10.1186/1475-925X-9-5

**Published:** 2010-01-15

**Authors:** Salvador A Melo, Bruno Macchiavello, Marcelino M Andrade, João LA Carvalho, Hervaldo S Carvalho, Daniel F Vasconcelos, Pedro A Berger, Adson F da Rocha, Francisco AO Nascimento

**Affiliations:** 1 Department of Electrical Engineering, University of Brasília, Brasília, DF, Brazil; 2 Department of Computer Science, University of Brasília, Brasília, DF, Brazil; 3 UnB-Gama Faculty, University of Brasília, Gama, DF, Brazil; 4 School of Medicine, University of Brasília, Brasília, DF, Brazil

## Abstract

**Background:**

Two-dimensional echocardiography (2D-echo) allows the evaluation of cardiac structures and their movements. A wide range of clinical diagnoses are based on the performance of the left ventricle. The evaluation of myocardial function is typically performed by manual segmentation of the ventricular cavity in a series of dynamic images. This process is laborious and operator dependent. The automatic segmentation of the left ventricle in 4-chamber long-axis images during diastole is troublesome, because of the opening of the mitral valve.

**Methods:**

This work presents a method for segmentation of the left ventricle in dynamic 2D-echo 4-chamber long-axis images over the complete cardiac cycle. The proposed algorithm is based on classic image processing techniques, including time-averaging and wavelet-based denoising, edge enhancement filtering, morphological operations, homotopy modification, and watershed segmentation. The proposed method is semi-automatic, requiring a single user intervention for identification of the position of the mitral valve in the first temporal frame of the video sequence. Image segmentation is performed on a set of dynamic 2D-echo images collected from an examination covering two consecutive cardiac cycles.

**Results:**

The proposed method is demonstrated and evaluated on twelve healthy volunteers. The results are quantitatively evaluated using four different metrics, in a comparison with contours manually segmented by a specialist, and with four alternative methods from the literature. The method's intra- and inter-operator variabilities are also evaluated.

**Conclusions:**

The proposed method allows the automatic construction of the area variation curve of the left ventricle corresponding to a complete cardiac cycle. This may potentially be used for the identification of several clinical parameters, including the area variation fraction. This parameter could potentially be used for evaluating the global systolic function of the left ventricle.

## Background

Two-dimensional echocardiography (2D-echo) allows the evaluation of cardiac structures and their movements, so that anatomic and functional characteristics may be assessed. The analysis of left ventricular function is extremely important in echocardiographic examinations, since a wide range of clinical diagnoses are based on the performance of this chamber [1]. This evaluation is typically performed by manual segmentation of the left ventricular cavity, in which a specialist delineates the blood-endocardial interface in a series of dynamic images. This process is laborious and operator dependent.

Several methods for automatic or semi-automatic segmentation of the left ventricular cavity in echocar-diographic images have been proposed in the literature. These methods are typically founded on image processing approaches. Klingler Jr *et al*. [2] proposed an image segmentation method using morphological operations. Choy and Jin used morphological filtering combined with edge enhancement filtering for segmenting the left ventricle in static images [3], and also used mathematical morphology and temporal information to improve the segmentation accuracy in sequences of dynamic 2D-echo images [4]. Ohyama *et al*. [5] used time-averaging combined with ternary quantization thresholding. Cheng *et al*. proposed a method using morphological operations and watershed segmentation for finding the center of the left ventricle [6], and then using snake deformation with a multiscale directional edge map for detecting the endocardial boundary [7]. Lacerda *et al*. [8] used a radial search algorithm based on temporal information for improving the segmentation quality. Dos Reis *et al*. [9] proposed a time-averaging denoising stage based on motion estimation for frame rejection. Amorim *et al*. [10] proposed a preprocessing stage based on image fusion to increase the accuracy of watershed segmentation. Fernández-Caballero and Vega-Riesco [11] presented a segmentation method using multi-stage preprocessing (smoothing, gradient detection, and gradient smoothing), a generalized Hough transform, and an active-contour algorithm. Despite all these efforts, there is currently no established gold standard for echocardiographic image segmentation. Ultrasound images are typically degraded by intensity inhomogeneity, distortion, and speckle noise. For these reasons, automatic segmentation of such images remains a challenging topic [12].

The segmentation of dynamic 2D-echo sequences could be particularly useful for detecting and locating wall motion abnormalities. The automatic segmentation of the left ventricle in 4-chamber long-axis images over the complete cardiac cycle is troublesome, because of the opening of the mitral valve during diastole. However, the information redundancy provided by the temporal component - e.g., periodicity of the cardiac cycle, similarity between adjacent temporal frames - may be exploited for reducing noise and/or avoiding undersegmentation [4,5,8].

This work presents a method for segmentation of the left ventricle in dynamic 2D-echo 4-chamber long-axis images over the complete cardiac cycle. The proposed algorithm is based on the combination and adaptation of techniques originally proposed by de Andrade *et al*. [13], associated with other classic 2D-echo image processing techniques [14]. These include time-averaging and wavelet-based denoising, edge enhancement filtering, morphological operations, homotopy modification, and watershed segmentation. The method combines these several established image processing approaches with a novel contour correction algorithm, and with the use of information from previous temporal frames for improved segmentation, including a new adaptive algorithm for addressing issues with mitral valve opening. The proposed method is semi-automatic, requiring a single user intervention for identification of the position of the mitral valve in the first temporal frame of the video sequence. Image segmentation is performed on a set of dynamic 2D-echo images collected from an examination covering two consecutive cardiac cycles. The segmentation of this set of dynamic images allows the automatic construction of the area variation curve (AVC) of the left ventricle corresponding to a complete cardiac cycle. This may potentially be used for the identification of several clinical parameters, including the area variation fraction (AVF). This parameter could potentially be used for evaluating the global systolic function of the left ventricle, which is important for diagnosing innumerous cardiovascular pathologies [1]. The proposed method for AVC and AVF calculation is demonstrated and evaluated on twelve healthy volunteers. The results are quantitatively evaluated using four different metrics, in a comparison with contours manually segmented by a specialist and with four alternative methods from the literature. The method's intra- and inter-operator variabilities are also evaluated.

## Methods

### Subjects and data acquisition protocol

The study involved 12 healthy individuals. Each subject went through a complete clinical examination and a 12-lead surface electrocardiogram evaluation. Individuals who presented symptoms of systemic cardiovascular problems or of cardiovascular disease were excluded from the study. The study was approved by the research ethics committee of the University of Brasília. Volunteers provided informed consent in accordance with institutional policy.

A dynamic image bank was constructed by specialists of the cardiology service of the hospital of the University of Brasília. The equipment used to obtain the dynamic 2D-echo images (Philips/ATL HDI 3500) was connected through an Ethernet network to a medical image server (GS-Web, Humano Tecnologia da Informação Ltda., Brasília-DF, Brazil), where the examinations were stored in a mass memory device for off-line processing.

Acquisitions corresponded to 12 video sequences (one per subject). Each sequence recorded two cardiac cycles of apical 4-chamber long-axis thoracic 2D-echo images, at a rate of 44 frames/s, totaling approximately 900 frames for the 12 individuals. The corresponding electrocardiographic signals were also acquired. A specialist evaluated the video sequences in terms of their image quality, using a score system ranging from 0 to 5 (Table 1). Sequences which scored 1 or lower were discarded (3 in total). Thus, a total of 9 video sequences were used for evaluating the proposed algorithm.

**Table 1:** Image quality of the video sequences.

Score	Quality	Number of sequences
0.5-1	very low	3 (discarded)
1.5-2	low	4
2.5-3	average	5
3.5-4	high	0
4.5-5	very high	0

The sequences were evaluated by a specialist in terms of their image quality, using a score system ranging from 0 to 5. Sequences which scored 1 or lower were discarded.

### Image segmentation algorithm

The construction of the area variation curve from the dynamic 2D-echo images was performed with a computational algorithm composed of two processing modules, which implement image preprocessing and segmentation algorithms, respectively. The preprocessing module improves image quality by reducing spurious noise (e.g., reverberation, mirroring). The second module performs the semi-automatic segmentation of the left ventricular cavity, and calculates the ventricular area.

**Figure 1:**
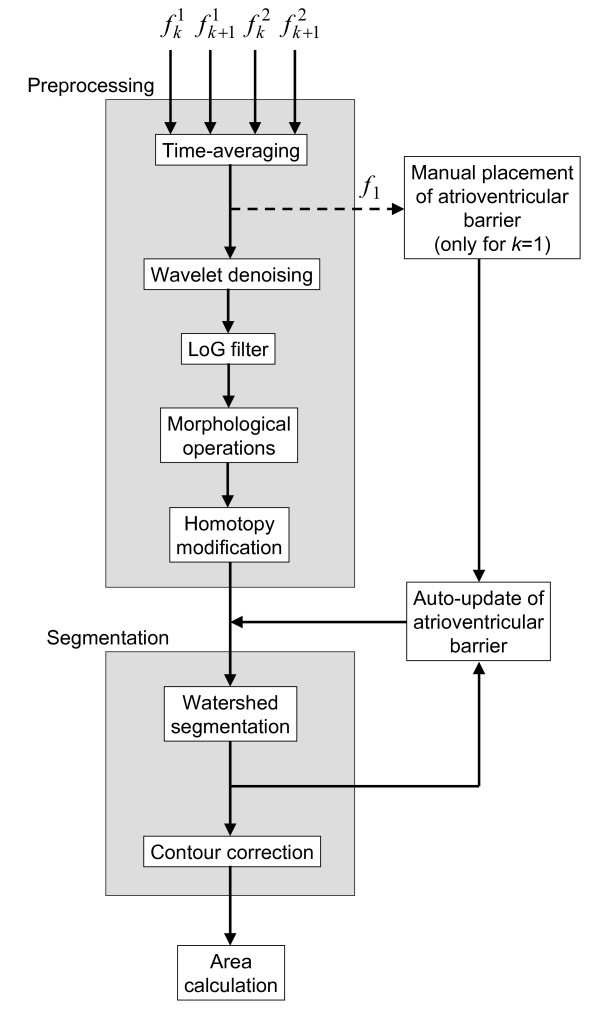
**Block diagram of the proposed algorithm**. The algorithm is composed of two image processing modules: preprocessing and segmentation. Four images - two adjacent images from two successive cardiac cycles - are used as inputs. The output is the area of the left ventricle at the corresponding time instant. The only operator interaction is a one-time-only manual placement of a barrier over the mitral valve.

A block diagram of the algorithm is shown in Figure 1. Four 2D-echo images - two adjacent images from two successive cardiac cycles - are used as inputs. The output is the area of the left ventricle at the corresponding time instant. The only operator interaction in the process is a one-time-only manual placement of a barrier over the mitral valve (discussed later).

#### Preprocessing module

The preprocessing module receives raw 2D-echo images (noisy, low contrast), and outputs images which are more adequate for segmentation. This module is implemented in five stages: time-averaging filter, wavelet-based denoising, edge enhancement, morphological operations, and homotopy modification.

##### Time-averaging filter

**Figure 2:**
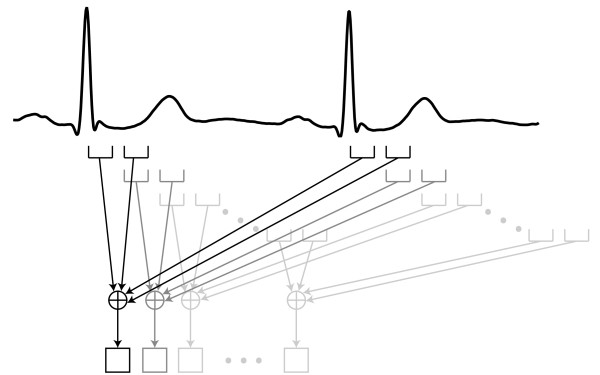
**Time-averaging process**. Four images from two consecutive cardiac cycles are averaged. The cycles are synchronized using the R wave of the electrocardiographic signal. A new composite image is obtained for each temporal frame within a cycle, using a sliding-window approach.

**Figure 3:**
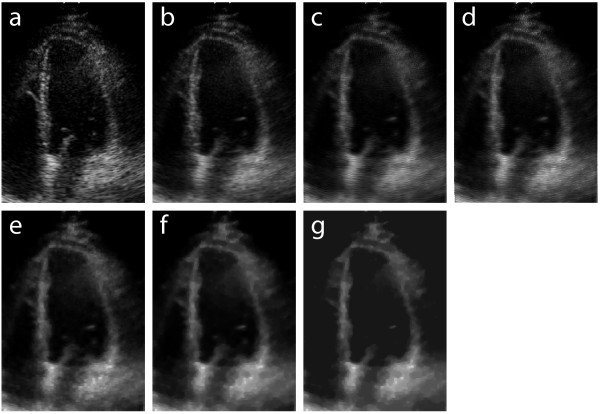
**Step-by-step preprocessing results**. (a) Input image (); (b) after time-averaging; (c) after wavelet denoising; (d) after edge enhancement; (e) after morphological closing and opening (disk radius = 1); (f) after morphological closing (disk radius = 3); (g) after homotopy modification. This temporal-frame corresponds to end-diastole.

In the first stage of the preprocessing module, a time-averaging filter [5,15] is applied for noise reduction. The temporal average is calculated for sets of four images taken from two consecutive cardiac cycles. Averaging is first performed for two successive images in each of the two cycles. Then, the images of the two cardiac cycles are synchronized based on the R wave of the electrocardiographic signal (Figure 2). The time-averaging filter is implemented as(1)

where  is the pixel value at spatial coordinates *x*, *y *in frame *k *of cycle *m*, and *f*_*k*_(*x, y*) is the resulting pixel value. This process assumes that successive images in the same cardiac cycle are highly correlated (given the inertia of the process), and that each image is highly correlated with its reciprocal in the previous cardiac cycle (given the phenomenon's periodicity). Although a new composite image is obtained every 23 ms (44 frames/s) using a sliding-window approach (see Figure 2), the time-averaging process reduces the effective temporal resolution from 23 ms to 46 ms. However, this is not expected to cause loss of diagnostic information, as cardiologists commonly use 40 ms resolution methods for electrocardiography, echocardiography, and magnetic resonance imaging -based diagnosis. In patients with irregular heart rate, arrhythmia rejection algorithms [16] may be used for exclusively selecting similar heartbeats. Alternatively, images from a single heartbeat may be used, at the cost of lower temporal resolution (by averaging additional adjacent frames) and/or inferior noise reduction performance. The effect of applying this time-averaging filter to the echocardiographic image shown in Figure 3(a) is demonstrated in Figure 3(b).

##### Wavelet-based denoising

The previous stage reduces white noise and enhances edges of interest by operating along the temporal dimension. This stage focuses solely on denoising, and operates along the spatial dimensions. The shift invariant wavelet denoising technique is used [17-19]. Daubechies wavelets have been shown to adapt to the characteristics of 2D-echo images, minimizing correlation in the transformed space, concentrating the energy of the information of interest, and adequately reducing white noise [20]. The 12th-order Daubechies wavelet was empirically chosen for this work, and provides a good representation in terms of mean squared error and transform-domain sparsity. Higher-order wavelets may be used with lower-quality images (e.g., non-echogenic subjects). This further removes noise, but reduces the spatial resolution. More detailed information on wavelet denoising is provided in Refs. [17-19]. This stage provides increased segmentation robustness in the presence of noise, but is computationally-intense, and may be considered optional for high-quality images. Figure 3(c) illustrates the effect of wavelet denoising on the image obtained from the time-averaging stage.

##### Edge enhancement

In the third stage of preprocessing, the images are filtered with a self-reinforcing Laplacian-of-Gaussian (LoG) filter. The LoG filter highlights the cavity edges by increasing image contrast, and improves the performance of the algorithm [3,4]. This self-reinforcing filter is implemented as(2)

where *m*(*x, y*) is the input image,  is the output image, , and * denotes two-dimensional convolution. Figure 3(d) illustrates the effect of the LoG filter on the image obtained from the wavelet denoising stage.

##### Morphological operations

This stage is used for non-linear smoothing. Morphological opening smoothes contours, breaks narrow isthmuses, and eliminates small islands and sharp peaks. Morphological closing smoothes contours, fuses narrow breaks and long thin gulfs, and eliminates small holes [21]. These procedures attenuate small local variations in image contrast, and help avoiding both undersegmentation and supersegmentation when the watershed algorithm is used. First, morphological closing is performed, followed by morphological opening (both using a disk of radius 1 as structuring element). The result of these operations is illustrated in Figure 3(e). Then, morphological closing is performed, using a disk of radius 3 as structuring element. The result is illustrated in Figure 3(f).

##### Homotopy modification

A homotopy modification [22] is used to reduce the number of local minima in the image and avoid supersegmentation when the watershed algorithm is applied. In this procedure, all local minima which are lower than a threshold are eliminated. For typical 2D-echo images that focus on the left ventricle, it is reasonable to assume that at least 50% of the pixels are part of the ventricular cavity. Thus, we use a threshold which is equal to the median of the histogram of the image obtained from the previous stage. The homotopy modification process consists in modifying the gradient function of the image in order to produce a new gradient. The minima of the gradient function are replaced by a set of markers [22]. Homotopy modification is especially useful for low-quality 2D-echo images (e.g., non-echogenic subjects), because such images present more irregular surfaces. Figure 3(g) illustrates the effect of homotopy modification on the image obtained from the previous stage.

#### Segmentation algorithm

The second module of the AVC construction system is the segmentation algorithm shown in the block diagram in Figure 1. This module is composed of three stages: insertion of atrioventricular barrier, watershed segmentation, and contour correction.

##### Atrioventricular barrier

**Figure 4:**
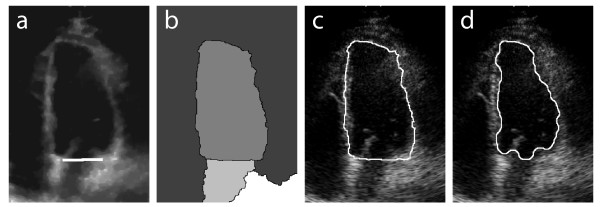
**Step-by-step segmentation results**. (a) Preprocessed image with atrioventricular barrier; (b) after watershed segmentation; (c) watershed contour; (d) corrected contour.

Before watershed segmentation, it is necessary to create a barrier between the left atrium and the left ventricle, over the entire extension of the mitral valve, as illustrated in Figure 4(a). Such barrier is necessary because of the opening of the mitral valve during diastole, when blood passes from the atrium into the ventricle. Without this barrier, the watershed algorithm would jointly segment part of the atrium during diastole. The atrioventricular barrier is manually prescribed for the first frame of the video sequence (Figure 1). In successive frames, the reallocation of the barrier becomes a totally automatic and adaptive process, which follows the movement of the mitral valve (see additional file 1). This tracking algorithm is controlled by the change in ventricular area along the cardiac cycle. Horizontally, the barrier continuously extends across the same *x*-coordinates as the originally prescribed barrier. Vertically, the updated barrier is placed on the *y*-coordinate of the lowest-positioned pixel in the previous frame's watershed mask (discussed next). The barrier is continuously updated during the entire cycle, but it generally has no effect when the mitral valve is closed.

In video sequences with segments of poor image contrast between muscle and blood, or in which the ventricular cavity was not fully covered by the acoustic beam, one additional barrier was manually placed to avoid undersegmentation (watershed "leakage"). This secondary barrier was placed as a tangent to the ventricular wall or to the beam's shadow, and was not dynamically updated.

##### Watershed segmentation

Watershed transformation [21,23,24] is used for image segmentation. The watershed algorithm can be defined as a process of flooding basins. Suppose that each local minimum of the image is treated as the deepest point of a basin. These basins are flooded from these points, and the water level rises gradually. To avoid a union between two basins, a dam is constructed at each point of contact. The unions of all the dams are the water partition lines. These lines represent the final segmentation. When applied to the preprocessed 2D-echo images, watershed segmentation results in an image containing several segmented regions, including the region of interest (inner wall of the left ventricle). This is illustrated in Figure 4(b). The region of interest is automatically selected, given its characteristic of being the largest central region.

##### Contour correction

**Figure 5:**
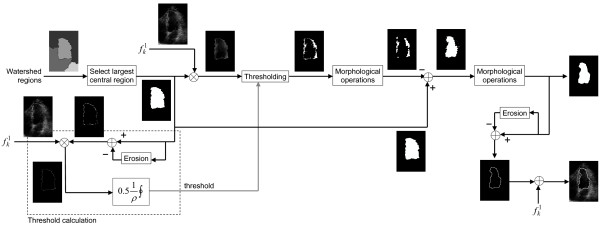
**Contour correction algorithm**. The largest central watershed region is used to mask the left ventricular wall and cavity. A threshold operation is used to separate myocardial muscle from blood pool, and morphological operations are used to produce a smoother mask. This is subtracted from the watershed mask, resulting in an estimate of the ventricular cavity, which is smoothed using additional morphological operations. Note: *ρ *is the perimeter of the contour (in number of pixels), and ∮ denotes a contour integral. Thus,  ∮ represents the mean pixel intensity along the contour.

The desired segmented contour should be placed exactly around the interface between blood and myocardium. However, the watershed algorithm typically produces a contour placed within the myocardial muscle, as illustrated in Figure 4(c). The true ventricular area is smaller than the watershed area, and the true blood-endocardial interface is contained within the watershed contour. Figure 5 presents a diagram of the proposed contour correction algorithm. The largest central watershed region is used to mask the left ventricular wall and cavity. Then, a threshold operation is used to separate myocardial muscle from blood pool. This threshold is determined by calculating the mean pixel intensity along the watershed contour, and multiplying this by a empirically-selected constant (0.5). Then, morphological closing, followed by morphological opening (both using a disk of radius 3 as structuring element), are used to produce a smoother mask. This is subtracted from the watershed mask, resulting in an estimate of the ventricular cavity. Finally, this mask is smoothed using morphological closing and opening (with a disk of radius 9). The final result is illustrated in Figure 4(d).

### Area variation curve and fraction

**Figure 6:**
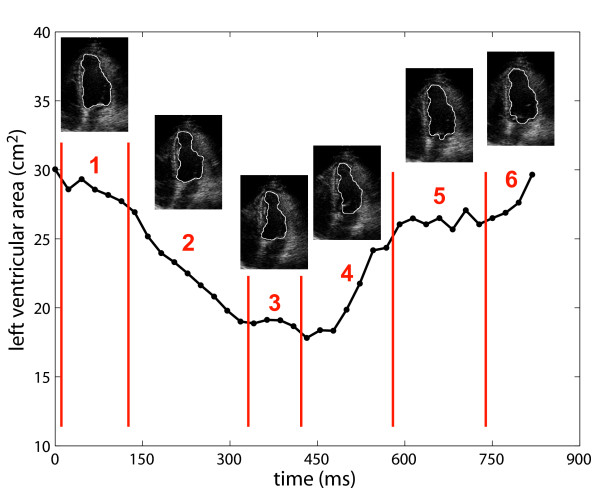
**Stages of the cardiac cycle in the typical AVC, as identified by a specialist**. (1) Isovolumetric contraction, (2) ejection, (3) isovolumetric relaxation, (4) rapid filling, (5) slow filling, and (6) atrial systole.

The left ventricular area associated with each temporal frame is calculated by counting the number of pixels contained within the associated segmented contour, and multiplying this count by the pixel area. The area variation curve is constructed by plotting the ventricular area as a function of time (Figure 6). The AVC is a representation of the left ventricular contractile function, and allows a concise evaluation of the cardiac cycle, allowing the identification of the various stages of the ventricular systolic and diastolic functions by the specialist.

From the AVC it is also possible to obtain the area variation fraction. The AVF is a measure of the variation of left ventricular area between systole and diastole. This may potentially be used for globally evaluating the left ventricular systolic function. The AVF provides diagnostic information similar to that of the ventricular shortening fraction, which is the percentage change in internal diameter from diastole to systole, and is a measure of global myocardial function. The AVF is computed as the percent difference between the end-diastolic area (*A*) and the end-systolic area (*E*) of the left ventricle (which correspond to the largest and smallest ventricular areas found during the cardiac cycle, respectively), as follows:(3)

The time instants associated with end-diastole and end-systole may be manually identified by the operator by inspecting the AVC and/or the 2D-echo images, or these may be automatically selected as the first frame after the R wave of the electrocardiogram and the frame corresponding to the lowest ventricular area, respectively.

### Qualitative and quantitative evaluation

The proposed method was implemented in Matlab (The MathWorks, Inc., Natick, MA, USA), using version 1.5 of the SDC Morphology Toolbox (SDC Information Systems, Naperville, IL, USA). The dynamic segmentation results were qualitatively evaluated, based on the calculated AVCs and AVFs, and also by visual inspection of the segmented images.

For a quantitative evaluation of the proposed method, we used an image bank composed of 60 echocar-diographic images, which had been previously manually-segmented by a specialist, and evaluated in terms of image quality (high, average, or low). Complete video sequences for these images were not available, thus an alternate implementation of the algorithm was used [13]. This implementation does not use the time-averaging and atrioventricular barrier stages, as these stages make use of additional temporal frames. The automatically-estimated ventricular contours were quantitatively compared with the manually-segmented contours, using four metrics: root mean squared distance (RMSd) [3], cross-correlation coefficient (CCC), percent error (PE) [25], and error sum (ES) [25], as follows.

The RMSd metric measures the root mean squared distance between paired contour points:(4)

where *L*_*A *_and *L*_*M *_are sets of points along the automatically-estimated and manually-segmented contours, respectively. Points along the longer contour are evenly eliminated, so that each contour has *N *points.

The distance in position between paired contour points -  and  - is considered. A more detailed description of this metric is found in Refs. [3] and [4].

CCC is a measure of similarity between the segmented regions, PE is a measure of the area estimation error, and ES is a measure of the edge-positioning error, as follows:(5)(6)(7)

where *M *and *A *are *m *× *n *binary images, associated with the manually-segmented and automatically-estimated contours, respectively, in which pixels within the contour (inclusive) are set to 1, and all other pixels are set to 0; |*X*| is the sum of all pixels in image *X*;  = *X *- *x*, where *x *is the mean value of image *X*, i.e., *x *= |*X*|/(*m *× *n*); *X*·*Y *denotes pixel-by-pixel image multiplication;  is the complement of image *X *(binary pixel inversion); and ∩ denotes intersection (i.e., binary pixel-by-pixel image multiplication).

### Intra- and inter-operator variabilities

The proposed semi-automatic method involves minimal manual intervention, since the location of the atrioventricular barrier is performed only for the first image of the sequence of frames. However, this intervention may lead to variations in measured areas, since the manually-placed barrier is interpreted by the algorithm as a border of the left ventricular endocardium. There are studies on the automatic location of the mitral valve [26,27]. These algorithms may totally eliminate user intervention, and may be easily incorporated to the proposed tool. However, mitral valve identification algorithms are computationally intense.

In order to evaluate the impact of the manual placement of the barrier, the following experiment was proposed. Four operators were asked to draw the atrioventricular barrier for the first frame of one of the video sequences (classified as of average image quality by a specialist). Each operator could not see the other operators' barriers. After each operator defined the barrier, the process was repeated in a circular fashion. A total of 40 barrier placements were performed for the same image (10 per operator). The operators could not see the segmentation results until all repetitions had been performed. The segmentation algorithm was then independently executed 40 times, using each barrier. The AVC associated with each barrier was calculated, and the mean area and standard deviation was calculated for each temporal frame.

## Results and Discussion

**Figure 7:**
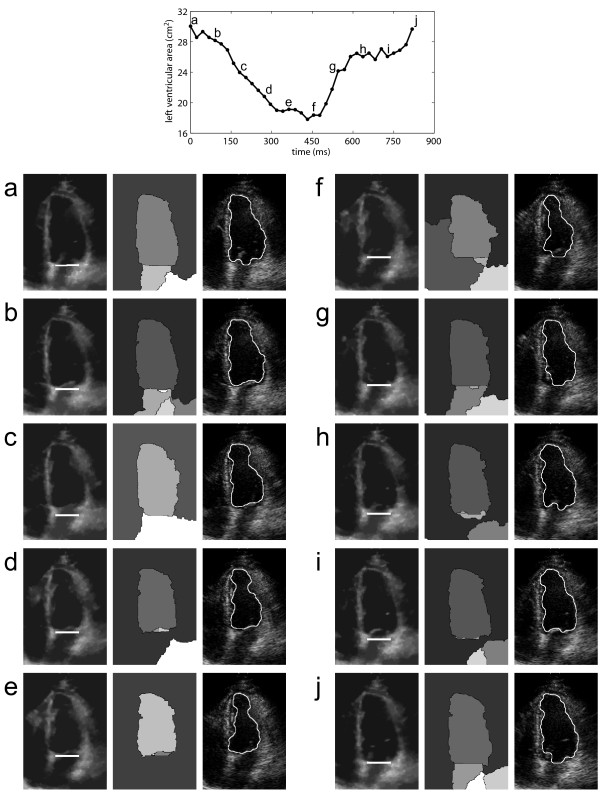
**Image segmentation results in ten temporal frames along a complete cardiac cycle**. The result for every 4th temporal frame is shown (a-j), as indicated on the measured AVC (top). For each temporal frame, it is shown: (left) preprocessed image with atrioventricular barrier; (center) watershed segmentation; (right) estimated contour. This 2D-echo sequence received a score of 2.5 out of 5 (average image quality) by a specialist.

A visual examination of the automatically segmented contours shows that the proposed method is capable of accurately delineating the left ventricular cavity in dynamic 2D-echo sequences (Figure 7). In images evaluated as of low quality, more significant segmentation errors were observed. However, such images are typically discarded by specialists and reacquired, as they provide reduced diagnostic reliability and are visually difficult to segment. Figure 7 demonstrates the algorithm performance for a sequence considered as of average image quality (score = 2.5), over a complete cardiac cycle. The images illustrate the dynamic positioning of the atrioventricular barrier (overlaid onto the preprocessed images), the watershed segmentation results, and the automatically-estimated contours (overlaid onto the original/unprocessed images). The opening of the mitral valve may be appreciated in the temporal frames associated with rapid filling - (f), (g), and (h) - and atrial systole - (a) and (j). In such frames, the barrier is noticeably used to delimitate the boundary between left atrium and left ventricle. The segmented images also confirm that the reduction in estimated area is caused mainly by its longitudinal reduction, as clinically expected. A video depicting all 37 frames of the cardiac cycle is also provided (additional file 1).

**Figure 8:**
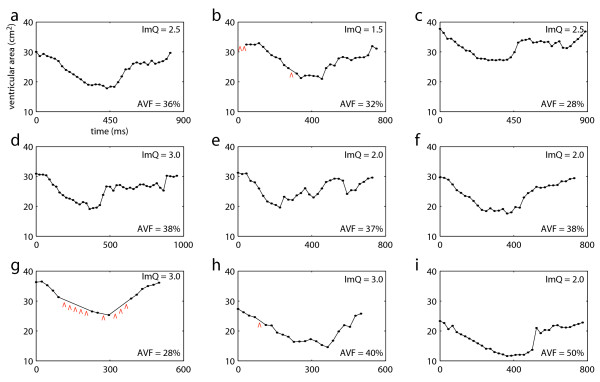
**Measured AVCs for nine subjects**. Red arrows in (b), (g), and (h) indicate temporal frames in which the automatic segmentation failed. The image quality score (ImQ) and measured AVF for each data set are indicated.

Figure 8 presents the measured AVCs for the selected video sequences. The red arrows in (b), (g), and (h) indicate temporal frames in which the automatic segmentation failed. These were mainly due to low contrast between muscle and blood, and due to acoustic shadowing. Severe segmentation issues due to rib artifacts were observed in sequences (e), (h) and (i). In these sequences, the measured ventricular areas in some (e) or all frames (h, i) were considerably lower then the true values. Nevertheless, all AVCs present a similar shape, distinctly alternating between a period of large area (diastole) and a period of small area (systole). These curves reflect the global wall motion in different stages of the cardiac cycle, from isovolumetric contraction to atrial systole (see Figure 6). Figure 8 also presents the measured AVF for each subject. Except for the result in (i), which was severely affected by image artifacts, all measured AVFs are within the typical range of AVF values in healthy subjects [1]. This is the expected result, as the selected subjects did not present cardiac pathologies.

Table 2 presents the results of the quantitative evaluation. The proposed method is compared with four alternative echocardiographic image segmentation algorithms from the literature. The proposed method achieved higher cross-correlation with manual segmentation than the methods by Klingler Jr *et al*. [2] and Amorim *et al*. [10] for high quality images. However, it is important to note that Klingler Jr *et al*. used composite images, each obtained by averaging over ten cardiac cycles, and that the quality of individual images is only described as "acceptable". Furthermore, their method was evaluated only on canine models. Compared with the results presented by Choy and Jin [3], the proposed method had lower performance in terms of RMSd for high quality images, but performed better for images of low and average image quality. The proposed method also presented better overall performance than the algorithm by Lacerda *et al*. [8], in terms of percent error and error sum.

**Table 2:** Quantitative comparison between the proposed approach and alternative methods from the literature.

	image quality	number of images	RMSd	CCC	PE (%)	ES (%)
Proposed method	high	20	2.14 ± 0.367	0.985 ± 0.011	2.49 ± 2.46	9.62 ± 7.9
	average	23	2.53 ± 0.398	0.901 ± 0.024	7.56 ± 2.51	16.20 ± 8.23
	low	17	2.99 ± 0.695	0.734 ± 0.101	15.10 ± 7.98	18.59 ± 7.02

Klingler Jr *et al*. [2]	acceptable^a^	121^b^		0.93		

Choy and Jin [3]	high	10	1.7 ± 0.7			
	average	8	3.5 ± 1.0			

Lacerda *et al*. [8]	high	7			10.65 ± 2.47	18.51 ± 5.06
	average	5			18.06 ± 7.49	24.62 ± 7.89

Amorim *et al*. [10]	high	8		0.917		10.79 ± 2.95

^a ^canine models;

^b ^composite images, each obtained by averaging over ten cardiac cycles.

The mean (± standard deviation) results from each evaluation metric are shown for different image segmentation methods and different degrees of image quality.

**Figure 9:**
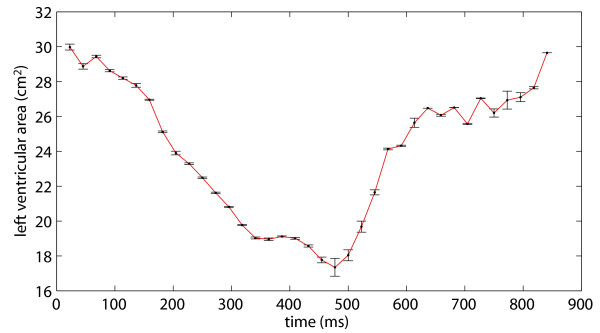
**Intra- and inter-operator variability of ventricular area estimation**. The atrioventricular barrier placement procedure was performed by four operators, with ten repetitions for each operator. This AVC shows the mean ventricular area for each temporal frame (dots), and the corresponding standard deviations (error bars). Measured AVFs ranged from 35.7% to 37.1% (36.2% ± 0.4%).

Figure 9 presents the results of the experiment for evaluating intra- and inter-operator variabilities. The atrioventricular barrier placement procedure was performed by four operators, with ten repetitions for each operator. The graph shows the mean ventricular area for each temporal frame, and the associated standard deviations. The standard deviations on each temporal frame ranged from 0% to 0.51%, with a mean of 0.12%. Measured AVFs ranged from 35.7% to 37.1%. The mean AVF was 36.2%, and the standard deviation was 0.4%. Such small variations between repetitions ensure the repeatability of the results generated by the algorithm. This indicates that user intervention does not generate undesired discrepancies when evaluating clinically important parameters, such as the AVF.

The watershed algorithm is a common technique for image segmentation, but its use in medical imaging has been limited, mostly due to oversegmentation and sensitivity to noise. In order to solve this problem, the watershed transformation has typically been used in combination with prior shape knowledge [28], or with contour extraction techniques [3]. In this work, a multi-stage preprocessing module was used to reduce image noise, increase image contrast, and modify the image homotopy. After preprocessing, the watershed algorithm is capable of successfully segmenting the left ventricle. The final contour is obtained by reducing the size of the segmented region, using a novel contour correction algorithm.

The proposed segmentation algorithm improves upon the method proposed by Andrade *et al*. [13] by incorporating a time-averaging denoising stage and an atrioventricular barrier stage. These stages make use of additional temporal frames for noise reduction and for addressing issues with mitral valve opening, respectively. As a result, the algorithm may now be used over a complete cardiac cycle, thus allowing the construction of the area variation curve and the calculation of the area variation fraction. The drawbacks of the proposed temporal-averaging stage are a reduction in temporal resolution (from 23 ms to 46 ms) and the need for data from two consecutive cardiac cycles. These are not expected to cause loss of diagnostic information, as the use of 50 ms temporal resolution and of acquisition segmentation across multiple heartbeats is widely accepted in several cardiovascular imaging modalities. The main limitation of the proposed atrioventricular barrier stage is the requirement of operator interaction. However, the proposed method may be made fully automatic by replacing this stage with an automatic mitral valve identification algorithm [26,27].

The proposed method for estimating the AVF could potentially be used for evaluating the global systolic function of the left ventricle. In patients with severe regional wall motion abnormalities, the AVF is expected to reflect segmental abnormalities and could potentially be used for distinguishing between normal and abnormal subjects. In patients with moderate or mild regional abnormalities, the proposed method could be used in combination with other approaches - e.g., ejection fraction, cardiac output, initial diastolic volume, atrial volume, mitral flow - for a more accurate diagnosis. A potentially useful technique for assessing regional wall function in echocardiography is color kinesis [29,30]. While the proposed method uses image processing techniques for identifying the blood-endocardial interface, color kinesis processes the ultrasound backscatter data. However, visualization and quantitation approaches similar to those used in color kinesis could potentially be implemented using the contours estimated with the proposed image segmentation method. Future work will be aimed at implementing estimation of regional AVF, for detecting and locating segmental wall motion abnormalities.

Only apical 4-chamber long-axis views were used in this work. However, the proposed technique may also be used in apical 2-chamber short-axis and long-axis images. The combined segmentation of these different views may potentially be used for estimating left ventricular volume [1]. From this, it would be possible to construct the volume variation curve, which may provide information complementary to the AVC. The proposed algorithm is expected to also work well with 2-chamber long-axis images, since these typically present similar visual features and image quality when compared with 4-chamber long-axis images. A similar algorithm for automatic segmentation of dynamic 2-chamber short-axis images is proposed in Refs. [9,31]. A methodology that aids medical professionals in diagnosing cardiac pathologies based on the area and/or volume variation curves may be a natural continuation of this study.

## Conclusions

We presented a semi-automatic image-segmentation algorithm for dynamically estimating the left ventricular area from two-dimensional echocardiographic video, and constructing the area variation curve over a complete cardiac cycle. The area variation fraction results obtained from the AVCs of healthy volunteers were in agreement with medical literature [1]. The AVC may be used by specialists for visually identifying the main stages of the cardiac cycle, such as atrial systole, rapid filling, slow filling, and ejection. The measurement of the duration of each of these stages may be useful for clinical analysis of global ventricular function. The AVF may potentially be used for functional evaluation of patients with different cardiopathies, especially those with severe functional repercussion.

## Competing interests

PAB, AFR and FAON declare potential ownership interests: Signax Engenharia e Tecnologia da Informação Ltda. HSC declares potential ownership interests: Humano Tecnologia da Informação Ltda. SAMJ, BM, MMA, JLAC and DFV have no competing interests.

## Authors' contributions

BM and MMA set up the experimental infrastructure, designed, implemented, and evaluated the original version of the proposed algorithm, and helped with text writing and quantitative evaluation. SAMJ improved the algorithm, wrote the first draft of this manuscript, and helped with video editing. JLAC and PAB helped with team management, technical consulting, experimental methodology, text structuring and writing, figure design, and algorithm refining. HSC provided clinical motivation and interpretation, and participated in the experimental setup and algorithm design. DFV acquired the echocardiographic data, manually segmented the images, and provided clinical evaluation of the results. AFR helped with algorithm design, and provided technical consulting and advising. FAON was the M.Sc. thesis advisor of BM, and is the Ph.D. thesis advisor of SAMJ. He is the principal investigator for the project that funded this work, and proposed and refined the segmentation algorithm. All authors read and approved the final manuscript.

## Supplementary Material

Additional file 1 **Video clip showing the dynamic segmentation of the left ventricle**. [MPEG2 video: addfile1.mpg] (left) preprocessed image with atrioventricular barrier; (center) watershed segmentation; (right) estimated contour. Note the automatic positioning of the atrioventricular barrier, which dynamically tracks the mitral valve. This 2D-echo sequence was classified as of average image quality by a specialist. Click here for file
